# Comment on “Risk factors for acute compartment syndrome of the leg associated with tibial diaphyseal fractures in adults” by Shagdan B et al.

**DOI:** 10.1007/s10195-015-0374-7

**Published:** 2015-09-01

**Authors:** Mark E. Hake, Cyril Mauffrey

**Affiliations:** Department of Orthopaedic Surgery, University of Michigan, 1500 E. Medical Center Dr, Ann Arbor, MI 48109 USA; Department of Orthopaedic Surgery, Denver Health Medical Center, 777 Bannock St, MC 0188, Denver, CO 80204 USA

The diagnosis of acute compartment syndrome (ACS) remains difficult and stress inducing despite continued efforts to define causes and risk factors of this condition. While a number of diagnostic techniques have been described [1, 2], ACS remains a clinical diagnosis. The patient’s history and physical exam findings reveal the earliest clues to the development of ACS, which evolves over time. This study by Shadgan and colleagues [3] confirms previous studies showing a lack of risk factors (other than age) that can help to heighten awareness of ACS in a subset of patients.

This study is the second largest of its kind looking at risk factors of acute compartment syndrome in tibial shaft fractures. The largest study was published in 2000 by McQueen [4] showing a higher risk of ACS in younger patients, as well as in men in their subset of patient with tibial shaft fractures. The present study differs from the earlier study in a number of ways. First, there was no difference found between men and women in rates of ACS. Second, the rate of ACS was higher (7.73 vs 4.3 %). This may reflect a true difference in how often ACS develops in tibial shaft fractures between two distinct populations. It also may be due to a difference in the rates of diagnosis as awareness may have been higher during the period from 1997 to 2011 than from 1988 to 1995 since many high-quality studies increased our knowledge on the topic of ACS.

The main benefit of the present study is to show that all patients with tibial shaft fractures should be monitored closely for ACS. The authors evaluated many potential risk factors including injury mechanism, fracture location, soft tissue status and method of fixation. The fact that none of these was found to be a statistically significant risk factor shows that the pathophysiology of ACS is such that it can develop in many different patients with variable presentations. While outcomes were not presented here, we know from other work that the consequence of a missed compartment syndrome or a lengthy delay in treatment can be devastating to the patient.

In summary, this study adds to the relatively small body of knowledge we have showing that compartment syndrome needs to be considered in every patient with a tibial shaft fracture, especially if they are young.
